# Use of a Low-fidelity simulator to improve trans-nasal fibre-optic flexible laryngoscopy in the clinical setting: a randomized, single-blinded, prospective study

**DOI:** 10.1186/1916-0216-42-35

**Published:** 2013-05-21

**Authors:** Michael W Deutschmann, Warren K Yunker, John J Cho, Meri Andreassen, Shari Beveridge, John Douglas Bosch

**Affiliations:** 1 Division of Otolaryngology Head & Neck Surgery, Department of Surgery, University of Calgary, 262-1632 14th Avenue NW, Calgary, Canada; 2 Division of Paediatric Surgery, Department of Surgery, University of Calgary, Calgary, Canada; 3 Department of Rehabilitation Services, Alberta Health Services, Calgary Zone, Calgary, Canada

**Keywords:** Endoscopy, Education, Simulation, Larynx

## Abstract

**Background:**

Trans-nasal flexible fibre-optic laryngoscopy (TFFL) is an essential skill for otolaryngologists. There is evidence to suggest that simulators help residents acquire procedural skills. The objective of this study was to examine the effect of simulation on endoscopy skill acquistion.

**Methods:**

A randomized controlled trial was conducted utilizing medical students and junior residents with limited experience in TFFL. Learners all performed a baseline endoscopy and were then randomized to receive either 45 minutes of simulation training or not. Following this, a second endoscopy was performed. Time to adequate visualization of the glottis, the percentage of time adequate visualization of the airway was maintained, and the number of collisions with mucosa were analyzed. Qualitative assessments were also obtained from the learner, patient, and staff laryngologist.

**Results:**

Time to adequate visualization of the glottis and the number of mucosal collisions were significantly less during the second endoscopy, irrespective of the use of simulation (84.8 sec vs. 68 sec, p < 0.01; 5.0 vs. 3.2, p < 0.01, respectively). Analysis using a two-way ANOVA with interaction established that none of the quantitative measures analyzed in this study improved with the addition of simulation.

**Conclusion:**

Improvements in time to visualization of the glottis and number of mucosal contacts were seen between the first and second endoscopy irrespective of simulator use. No additional benefit was conferred with the use of a low-fidelity simulator.

## Background

Trans-nasal fibre-optic flexible laryngoscopy (TFFL) is routinely performed by otolaryngologists, both at the bedside and in the out-patient setting. This procedure allows for detailed evaluation of the nasal, pharyngeal, and laryngeal anatomy. Prior to learning how to correctly recognize pathology, trainees must learn to manipulate the endoscope and pass it through the upper aerodigestive tract.

Surgical education has traditionally been based on the apprentice model, wherein skills are fostered and developed in the clinical setting [1]. Training involves first observing a trained surgeon perform the procedure and then having the student perform that same procedure on a patient. Although this process is integral to surgical education, the perception that patients are the first and only tool in surgical education has changed. In the modern healthcare environment, there is increasing pressure on medical educators to increase both the efficiency of medical education and the acquisition of procedural and surgical skills. In part, this has lead to the development and use of procedural simulators. Procedural simulators are any environment or situation, apart from the actual clinical setting, designed to foster the acquisition or development of a specific skill set. This allows the learner to acquire skills in a safe and low stress environment prior to practicing on a patient [2]. There are a variety of different simulators, including, but not limited to, extant animal models, human cadavers, and both low-fidelity and high-fidelity bench-top models. High-fidelity simulators are designed to enhance skills while providing a realistic procedural environment. Low-fidelity simulators are those that are designed to enhance specific skills without accurately recreating the procedural environment. Low-fidelity simulators have been shown to be useful in the acquisition of laparoscopic skills [3]. Similarly, it has been shown that medical students who trained on a TFFL high-fidelity simulator did significantly better when tested against their untrained peers on the same simulator [4]. Interestingly, this did not translate into shorter endoscopy times or improved patient comfort when using a standardized patient [4]. To date, there have been no studies examining the impact of low-fidelity simulator training on trans-nasal endoscopy in the clinical setting.

Our objective was to examine the effect of low-fidelity simulation training on TFFL skills in the clinical setting. To that end, we developed a low-fidelity TFFL simulator that allowed learners to practice endoscopic manipulation skills outside of the clinical setting. We subsequently designed an experiment to examine the effect of low-fidelity simulator training on clinical endoscopy skills. Our primary outcome measure was the time taken to insert the endoscope, traverse the nasal cavity and upper aerodigestive tract and visual the glottis.

## Methods

### Experimental design

Ethical approval was obtained from the University of Calgary Conjoint Health Research Ethics Board. The study was conducted through the Voice Disorders Clinic at the Rockyview General Hospital in Calgary, Alberta, a tertiary referral center for laryngeal disorders. A fellowship-trained laryngologist (JDB) supervised and evaluated all endoscopies. Learners were recruited from volunteer medical students and junior residents on otolaryngology rotations through the University of Calgary. All learners completed a questionnaire assessing prior endoscopy experience. All patients who presented to the Voice Disorders Clinic routinely require laryngeal examination and were solicited for their participation. All patients who underwent TFFL in this study were volunteers, with informed consent being obtained prior to endoscopy.

All learners observed one TFFL being performed by the senior author and received instruction on how to hold and manipulate the endoscope prior to performing an endoscopy. The learner then performed a TFFL on a clinic patient. During this endoscopy the senior author only provided guidance if required to ensure patient safety.

**Figure 1:**
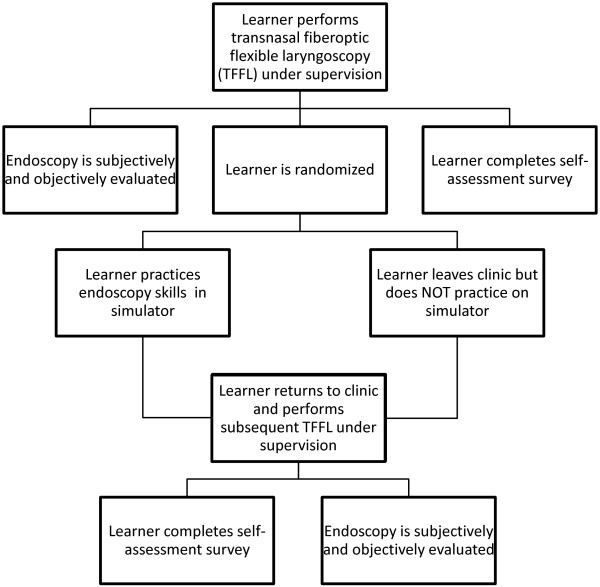
Study design.

Once the first endoscopy was complete, the learner was assigned to either the simulation-group or the control-group by random draw. Learners randomized to the simulation group were given 45 minutes to practice basic endoscopy skills using the low-fidelity simulator, whereas the control group was not. To ensure that the senior author remained blinded, learners from both groups were excused from clinic for 45 minutes. After the allotted time, all learners returned to clinic and performed a second TFFL. The second TFFL was on a different patient than their initial endoscopy (Figure 1).

In total, each learner performed two separate TFFLs. The laryngologist determined which side of the nose presented the best avenue for endoscopy. Topical intra-nasal lidocaine (20 mg/mL) was used for every endoscopy (AstraZeneca UK Ltd; London, UK). Nasal decongestant was not used. Lubricant was applied to the endoscope prior to insertion (Cardinal Health; Dublin, OH, USA). A distal chip endoscope (Pentax VNL 1170 K; Montvale, NJ, USA) was used for all endoscopies. All endoscopies were recorded for subsequent analysis.

The patient was blinded to which endoscopy number and group the learner was randomized to. The senior author was blinded to which study group the learner belonged to, but was not blinded to whether this was the learner’s first or second endoscopy. The endoscopy videos were later analyzed by one of the study investigators (MWD) who was blinded to randomization group, learner name, and whether it was the learner’s first or second endoscopy.

### Variables assessed

The variables assessed included: (1) time to adequate visualization of the glottis; (2) percentage of time adequate visualization of the airway was maintained during passage of the endoscope; and (3) total number of endoscope collisions with mucosa. Time to adequate visualization was defined as the amount of time required for the learner to pass the endoscope from the nasal alae until adequate visualization of the glottis was achieved. The percentage of time adequate visualization was maintained was defined as the percentage of time an adequate view of the airway was maintained, such that a structure within the nasal cavity or larynx could be readily identified rather than obscured by mucosa.

During both endoscopies the learner was also subjectively evaluated by the senior author (JDB) as well as the patient. Patient comfort, ease of manipulating the endoscope, and learner comfort were assessed using a 10-point scale, with 1 being strongly agree and 10 being strongly disagree. The learners also rated their own ability using the same scoring system.

### Low-fidelity simulator design

**Figure 2:**
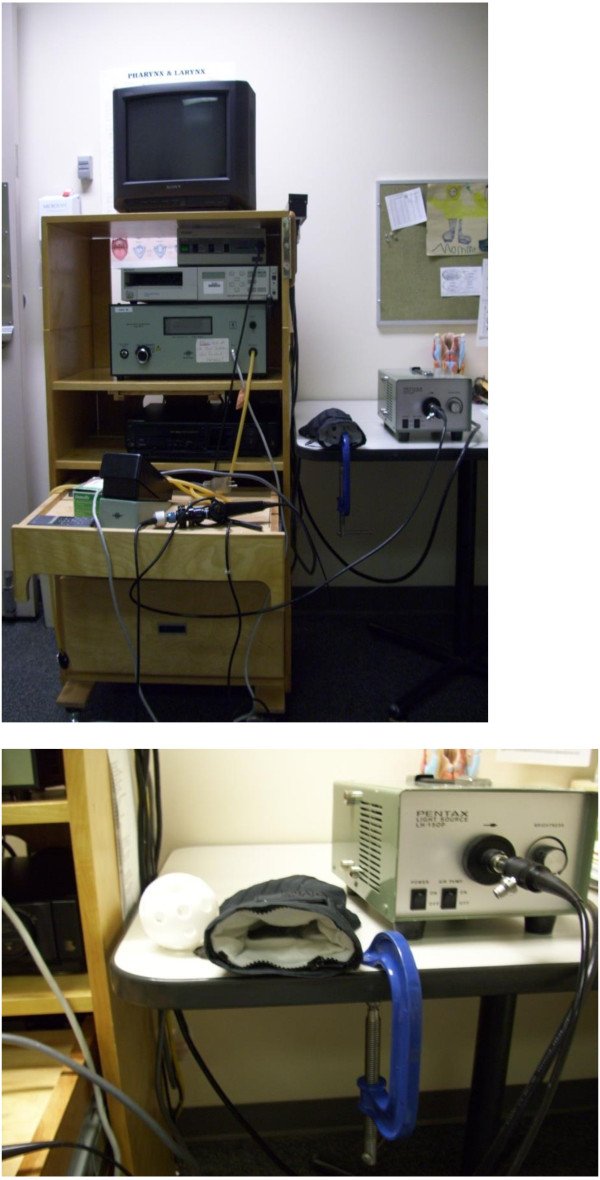
Image of simulator.

The simulator consisted of two parts: (1) a flexible endoscope attached to a digital camera and light source; and (2) a hollow plastic ball with multiple fenestrations located inside a glove (Figure 2). Learners were instructed to practice guiding the endoscope through the narrow passages in the ball and into the fingers of the glove.

### Data analysis

All data are presented as mean ± standard deviation (SD). An *a priori* power calculation was performed. Based on previous studies, a difference of 27.5 seconds in time to adequate visualization of the glottis was considered clinically relevant [4,5]. Combined with an α = 0.05 and a power of 0.81, 32 subjects per group were required. The effect of repeat endoscopy and simulation were compared separately using a *t*-test or its non-parametric equivalent. A two-way ANOVA with interaction was used to explore the effect of simulation, and repeat clinical endoscopy.

## Results

**Figure 3:**
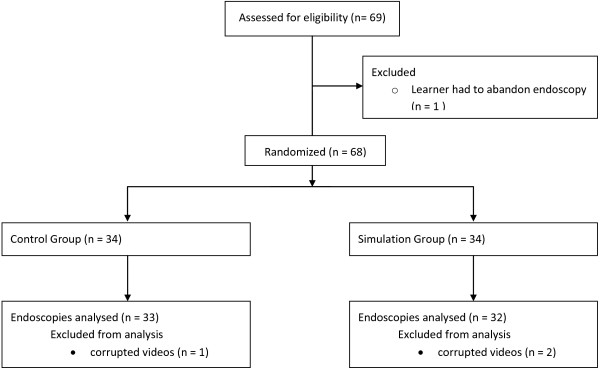
Flow of learners who participated in the study.

In total 69 learners were enrolled in the study. Four learners’ data sets were removed because they were incomplete. In three cases, the video files were corrupt, and in one instance the learner was unable to complete the endoscopy and, for the sake of patient comfort and safety, the principle investigator completed the endoscopy. The result was 32 learners in the simulation group and 33 in the control group (Figure 3).

Both groups had an equivalent amount of endoscopy experience prior to the study (Table 1). The first endoscopy of the control group was not significantly different than the first endoscopy of the simulation group for the three quantitative variables analyzed (Table 2; Column A vs. Column C; *p* value not shown). Similarly, the senior author’s assessment of patient comfort during the examination did not differ between the first endoscopy of the simulation and control groups (Table 2; Column A vs. Column C; *p* value not shown). In addition, there was also no difference in learners’ comfort and their ability to manipulate the endoscope as assessed by the senior author (Table 2; Column A vs. Column C; *p* value not shown). There was also no difference in patient comfort and willingness to repeat the examination, as assessed by the patients (Table 2; Column A vs. Column C; *p* value not shown). In summary, there was no qualitative or quantitative difference between the simulation and control groups’ first endoscopy.

**Table 1:** Learner demographics

**Number of previous trans-nasal fibre-optic laryngoscopies**	**Control group**	**Simulation group**	*p *value*
0-5	24	25	0.77
> 5	9	7	

* Fischer’s exact test.

**Table 2:** Analysis by group and order of endoscopy

	**Control group**		**Simulation group**		
**Variable**	**First endoscopy mean ± SD (CI) Column A**	**Second endoscopy mean ± SD (CI) Column B**	**First endoscopy mean ± SD (CI) Column C**	**Second endoscopy mean ± SD (CI) Column D**	**ANOVA *p *value**
Time to adequate visualization of the glottis (sec)	92.9 ± 42.60 (78.3 - 107.4)	65.6 ± 30.4 (55.2 - 76.0)	76.7 ± 32.7 (65.5 - 87.8)	70.4 ± 37.0 (57.8 - 83.0)	0.10
Number of mucosal contacts	5.2 ± 4.4 (3.7 - 6.7)	3.2 ± 3.2 (2.1 - 4.3)	4.9 ± 4.9 (3.2 - 6.6)	3.3 ± 2.8 (2.3 - 4.2)	0.77
Percentage of time adequate visualization of the airway was maintained	82.9 ± 16.3 (77.3 – 88.4)	82.4 ± 21.5 (75.1 – 89.8)	77.8 ± 22.4 (70.2 – 85.5)	83.4 ± 17.7 (77.3 – 89.4)	0.38
Learner comfort (self reported)*	4.6 ± 2.0	5.5 ± 1.9	3.9 ± 1.9	5.9 ± 2.0	0.13
Patient comfort (self reported)*	3.27 ± 2.4	3.1 ± 3.0	3.6 ± 3.1	2.9 ± 2.5	0.62
Patient willingness to repeat the examination*	1.6 ± 2.4	1.6 ± 2.9	1.7 ± 2.7	1.5 ± 2.4	0.84
Learner manipulation*	3.6 ± 2.7	5.3 ± 2.7	3.2 ± 2.3	5.9 ± 2.0	0.23
Learner comfort (investigator evaluation)*	3.5 ± 2.5	5.3 ± 2.5	3.1 ± 2.4	5.9 ± 2.2	0.24
Patient comfort (investigator evaluation)*	6.5 ± 2.1	7.1 ± 2.1	6.4 ± 2.5	7.1 ± 2.0	0.92

* = 1 to 10 scale; SD = standard deviation; CI = 95% confidence intervals.

Thirteen endoscopies (10% of the total) were randomly selected and re-analyzed by the same study investigator (MWD). The intraclass correlation coefficient (ICC) was used to calculate intra-rater reliability of the 3 main objective variables. A ICC of 0.7-0.8 indicates strong agreement and a ICC of > 0.8 indicates almost perfect agreement. The ICCs for time to adequate visualization of the glottis, percentage of time adequate visualization of the airway was maintained, and the total number of endoscope collisions with mucosa were 0.99, 0.76, and 0.81 respectively.

### *First* vs. *Second endoscopy*

Overall, there was a significant improvement both in the time to adequate visualization of the glottis and number of mucosal contacts between the first and second endoscopies, irrespective of simulator use (84.8 ± 38.0 sec vs. 68.0 ± 33.9 sec, p < 0.01; 5.0 ± 4.6 vs. 3.2 ± 3.0, p < 0.01; Table 3). There was no significant difference between the first and second endoscopy with respect to the amount of time adequate visualization was maintained (80.3 ± 19.6% vs. 82.9 ± 19.7%, p = 0.45; Table 3). Qualitatively, learners felt like they were more comfortable during the second endoscopy compared to the first (4.2 ± 1.9 vs. 5.7 ± 2.0, p < 0.01; Table 3). Similarly, the senior author felt that the learners were better, and more comfortable, at manipulating the scope during the second TFFL (3.4 ± 2.5 vs. 5.6 ± 2.4, p < 0.01; 3.3 ± 2.4 vs. 5.6 ± 2.4, p < 0.01; Table 3). However, patient comfort, graded by the senior author, did not vary between the first and second endoscopy (6.4 ± 2.4 vs. 7.1 ± 2.1, p = 0.09; Table 3). Similarly, from the patients’ perspective, there was no difference in comfort or willingness to repeat the examination (3.4 vs. 3.0, p = 0.38; 1.7 vs. 1.5, p = 0.79; Table 3).

**Table 3:** Analysis by order of endoscopy

**Variable**	**First endoscopy mean ± SD**	**Second endoscopy mean ± SD**	***p *value**
Time to adequate visualization of the glottis (sec)	84.8 ± 38.0	68 ± 33.9	< 0.01
Number of mucosal contacts	5.0 ± 4.6	3.23 ± 3.0	< 0.01
Percentage of time that adequate visualization of the airway was maintained	80.3 ± 19.6	82.9 ± 19.7	0.38
Learner comfort* (self reported)	4.2 ± 1.9	5.7 ± 2.0	< 0.01
Patient comfort* (self reported)	3.4 ± 2.8	3.0 ± 2.8	0.38
Patient willingness to repeat the examination	1.7 ± 2.6	1.5 ± 2.7	0.79
Learner manipulation (investigator evaluation)*	3.4 ± 2.5	5.6 ± 2.4	< 0.01
Learner comfort (investigator evaluation)*	3.3 ± 2.4	5.6 ± 2.4	< 0.01
Patient comfort (investigator evaluation)*	6.4 ± 2.4	7.1 ± 2.1	0.09

* = 1 to 10 scale; SD = standard deviation.

### *Simulation* vs. *Control group*

Data from the simulation group and control group, irrespective of the first and second endoscopy were compared. No significant differences in any of the quantitative or qualitative measures described were identified (Table 4).

**Table 4:** Analysis by study group

**Variable**	**Control group mean ± SD**	**Simulation group mean ± SD**	***p *value**
Time to adequate visualization of the glottis (sec)	84.8 ± 38.0	68 ± 33.9	0.36
Number of mucosal contacts	5.0 ± 4.6	3.23 ± 3.0	0.88
Percentage of time that adequate visualization of the airway was maintained	80.3 ± 19.6	82.9 ± 19.7	0.55
Learner comfort* (self reported)	4.2 ± 1.9	5.7 ± 2.0	0.59
Patient comfort* (self reported)	3.4 ± 2.8	3.0 ± 2.8	0.95
Patient willingness to repeat the examination	1.7 ± 2.6	1.5 ± 2.7	1.0
Learner manipulation (investigator evaluation)*	3.4 ± 2.5	5.6 ± 2.4	0.83
Learner comfort (investigator evaluation)*	3.3 ± 2.4	5.6 ± 2.4	0.83
Patient comfort (investigator evaluation)*	6.4 ± 2.4	7.1 ± 2.1	0.92

* = 1 to 10 scale; SD = standard deviation.

### Effect of simulation

A two-way ANOVA with interaction was used to explore the effect of both simulation and endoscopy. No interaction between simulation and repeat endoscopy was detected. Time to adequate visualization of the glottis, the number of mucosal contacts, and the percentage of time adequate visualization of the airway were maintained were not affected (Table 2).

## Discussion

In this study we examined the effect of low-fidelity simulation training, as well as the interaction of repeat clinical endoscopy and simulation training, on both clinical endoscopy skills and patient comfort. Overall, there was a significant reduction in time to adequate visualization and number of mucosal contacts between first and second endoscopy. This suggests that the benefits of repetition quickly become appreciable. A recent study has shown that learners become competent after 6 endoscopies [6]. Our study is in keeping with this notion of rapid skill acquisition with repeat endoscopy.

Interestingly, when data were stratified by both endoscopy number and simulation, there was no significant interaction seen. This suggests that, in this study, there was no additional benefit, above and beyond repeat endoscopy, conferred by the use of the simulator.

Learners performed TFFL on different patients for each endoscopy. While patients may have mild variations in their anatomy, none had had previous endoscopic sinus surgery, major head and neck surgery or head and neck external beam radiotherapy. Using different patients for the first and second endoscopy was important to prevent any improvement on the second endoscopy being attributable to familiarity with the patient’s anatomy.

In our study, we compared the second endoscopy following randomization with the baseline endoscopy. We did not analyze the subsequent endoscopies beyond this. It is possible, and would be interesting to examine, if the rate of skill acquisition may have been faster in the simulation group compared to the control group if subsequent endoscopies had been analyzed.

Based on our observations, the most challenging aspect of the endoscopy was navigating the nasal cavity. This anatomy was not reflected in the design of the simulator. It may be that, in this case, a simulator that better reflected nasal anatomy would be more beneficial. However, a randomized controlled study looking at rigid and flexible nasal endoscopy utilizing an accurate nasal model also did not show any quantitative or qualitative difference among simulator and control groups when they subsequently performed endoscopies on standardized patients [4]. This is likely due to the inherent challenges of performing endoscopy on a live patient rather than an inanimate object and could also help explain why our study did not detect a significant benefit with simulation. Developing a simulator that not only replicates the anatomy, but provides the ability to mimic a patient’s responses when undergoing endoscopy, may better address this issue.

## Conclusion

The aim of this study was to assess the effect of a low-fidelity simulator on endoscopy skills and patient comfort. Overall, we demonstrated that repeat endoscopy in the clinical setting had a significant impact on time to adequate visualization of the glottis and the number of mucosal contacts, but that no additional benefit was conferred with the use of a low-fidelity simulator. In the modern medical education environment, there is considerable focus on the use of simulation as a means of acquiring technical skills. While this can most certainly be a valuable education tool, not all skills require simulation training. We believe that this study demonstrates this fact and establishes that flexible trans-nasal endoscopy is such a skill.

## Competing interests

The authors declare that they have no competing interests.

## Authors’ contributions

MD was responsible for data collection and analysis, as well as drafting the manuscript. WY was responsible for the design of the study, performing the statistical analysis, and editing the manuscript. JC was responsible for data analysis and drafting the manuscript. MA was responsible for the design of the study, acquisition of data, and editing the manuscript. BS was also responsible for the design of the study, acquisition of data, and editing the manuscript. JB conceived the study, participated in its design and coordination and edited the manuscript. All authors read and approved the final manuscript.
